# A dataset of calcareous nannoplankton and smaller benthic foraminifera from a middle Eocene nummulitic accumulation (Transylvanian Basin, Romania)

**DOI:** 10.1016/j.dib.2021.107154

**Published:** 2021-05-15

**Authors:** Raluca Bindiu-Haitonic, Ramona Bălc, Szabolcs-Attila Kövecsi, George Pleș, Lóránd Silye

**Affiliations:** a Department of Geology and Center for Integrated Geological Studies, Babeş-Bolyai University, M. Kogălniceanu 1, 400084 Cluj-Napoca, Romania; b Faculty of Environmental Sciences and Engineering, Babeș-Bolyai University, Fântânele 30, 400084, Cluj Napoca, Romania; c Interdisciplinary Research Institute on Bio-Nano-Sciences, Babeş-Bolyai University, Treboniu Laurian 42, 400271, Cluj Napoca, Romania

**Keywords:** Calcareous nannoplankton, Smaller benthic foraminifera, Nummulitic accumulation, Paleoecology, Eocene

## Abstract

The dataset presents quantitative information on the taxa distribution of calcareous nannoplankton and smaller benthic foraminifera retrieved from the nummulitic accumulation in the Transylvanian Basin (Romania). Until now, only the larger benthic foraminifera and bryozoans were systematically studied, data on calcareous nannoplankton and smaller benthic foraminifera are scares. Our data comprise of quantitative information on the calcareous nannoplankton and smaller benthic foraminifera (raw individuals counts, relative abundance), diversity indices, benthic foraminifera dissolved oxygen index – BFOI and normalised data (ARCSIN values) used for clustering and principal component analysis. These data provide information on paleoenvironmental changes in the north-western part of the Transylvanian Basin during the middle Eocene (Bartonian) and can be used in any comparative study on the reconstruction and paleoenvironmental interpretation of the nummulitic carbonate reservoirs from Tethyan regions. The present data article is associated with the research paper “In the shadow of giants: Calcareous nannoplankton and smaller benthic foraminifera from an Eocene nummulitic accumulation (Transylvanian Basin, Romania)” by Bindiu-Haitonic et al. 2021 [1].

**Specifications Table**

**Table:** 

Subject	Geology
Specific subject area	Micropaleontology, middle Eocene (Bartonian) paleoenvironmental reconstruction based on calcareous nannoplankton and smaller benthic foraminifera
Type of data	Spreadsheets
How data were acquired	Calcareous nannoplankton: Light microscope (Axiolab A) with 1000 × magnification, the standard smear slide technique, AxioCam ERc5s digital microscopy camera, PAlaeontological STatistics (PAST) statistical package version 3.26b. Smaller benthic foraminifera: Binocular microscope (Optika, SZO-T+SZ-A1+SZ-ST8), SEM (Hitachi 8230), ASC Micro Sample Splitter, standard micropaleontological method (63 µm mesh sieve), and PAlaeontological STatistics (PAST) statistical package version 3.26b.
Data format	Raw Analyzed Filtered
Parameters for data collection	Except for the clay samples, the analyzed sediment was separated from the sandy clayey matrix that loosely binds the *Nummulites* specimens. The calcareous nannoplankton was recovered with the standard smear slide technique of Bown and Young [2]. The smaller benthic foraminifera, each sample was wet sieved through 63 µm and dried at 50 °C. The sandy samples were treated with 10%m, and then with 3% hydrogen peroxide solution.
Description of data collection	Calcareous nannoplankton: at least 300 specimens per slide were counted. Samples containing less specimens were analyzed in 800 different fields of view. Smaller foraminifera were hand-picked from the >63 µm residue, taxonomically identified, and counted under the stereomicroscope. Samples’ residue was split into aliquots of about 200–300 specimens.
Data source location	Twenty-one exposures from nine locations of the Transylvanian Basin, Romania. The location of the studied sections and the corresponding GPS points are presented in Table 1.
Data accessibility	Repository name: Mendeley Data Direct URL to data: http://dx.doi.org/10.17632/hsj6hrysxh.2
Related research article	Bindiu-Haitonic, R., Bălc, R., Kövecsi, Sz.A., Pleș, G., Silye, L., 2021. *In the shadow of giants: Calcareous nannoplankton and smaller benthic foraminifera from an Eocene nummulitic accumulation (Transylvanian Basin, Romania)*, Marine Micropaleontology 165, 101988, https://doi.org/10.1016/j.marmicro.2021.101988, [1].

**Value of the Data**

• The type of micropaleontological information presented in this article has not been provided in any existing regional databases so far.
• The quantitative data for calcareous nannoplankton and smaller benthic foraminifera could support future research of reconstructing paleoenvironmental changes in Tethyan regions during the middle Eocene.
• These data demonstrate that the diversity within a nummulitic accumulation is higher than expected before.
• The dataset in the article provides information for micropaleontological community, who can use and compare these results with their own.

## 1 Data Description

Data reported herein, have been obtained from the micropaleontological analysis of the middle Eocene (Bartonian) calcareous nannoplankton and smaller benthic foraminifera record from nine representative areas located in the Gilău (GSA) and Meseș (MSA) area from the Transylvanian Basin (Romania). The GPS coordinates are provided for each section (Table 1).

**Table 1:** Location of the studied outcrops grouped on localities.

		GPS coordinates	
Locality	Outcrop	Latitude	Longitude
Florești	FCF	46°44′00.0″	23°29′03.3″

Luna de Sus	Lu1 and Lu2	46°44′13.0″	23°26′06.8″
	LuR	46°43′41.47	23°25′44.89

Gilău	Gi1	46°46′00.4″	23°23′18.5″
	Gi2	46°46′18.8″	23°23′00.7″
	Gi3	46°46′19.7″	23°23′03.2″
	GiA	46°47′4.19″	23°23′18.42″

Căpușu Mare	Ca1	46°48′37.4″	23°14′28.5″
	Ca2	46°48′37.9″	23°14′28.7″

Leghia	Le1	46°50′31.00″	23°10′16.00″

Mănăstireni	M	46°47′25.0″	23°06′00.5″

Mănăstireni - Văleni	MV1	46°46′47.2″	23°03′16.9″
	MV2	46°46′48.2″	23°03′15.9″
	MV3	46°46′51.6″	23°03′09.8″
	MV4	46°46′54.4″	23°03′04.7″

Văleni	Va1	46°46′58.1″	23°02′57.6″
	Va2	46°47′10.0″	23°00′58.5″
	Va3 and Va4	46°46′43.43″	23° 3′4.64″

Rona	Ro	47°15′22.6″	23°17′31.8″

The micropaleontological data comprise raw species counts (Tables 1, 2 in [3]), relative abundances (Tables 3, 4 in [3]) and ARCSIN values used to perform multivariate analysis (Tables 5, 6 in [3]) for both calcareous nannoplankton and smaller benthic foraminiferal assemblages. The calculated abundance data of taxa, groups and microhabitats, the calculated benthic foraminifera dissolved oxygen index – BFOI (Table 7 in [3]) and diversity indices for smaller benthic foraminifera (Table 8 in [3]) are also included. Same background color in each table represents the same outcrop and the samples are stored in case of each outcrop in stratigraphic order (samples with the same style – italic and bold – belong to the same stratigraphic level). In the case of the calcareous nannoplankton datasets (Tables 1, 3, 5 in [3]) the samples written with red had less than 50 specimens and they were excluded from the statistical analysis. These data records are separately available as spreadsheets files (.xlxs) at Mendeley data [3].

## 2 Experimental Design, Materials and Methods

The studied sedimentary record was sampled between 2014 and 2019 during various field campaigns carried out in the north-western part of the Transylvanian Basin (GSA and MSA, Romania). The samples were retrieved from 21 outcrops, located around 9 localities (Table 1). The number of samples collected at one outcrop is 2 to 9 depending on the height and lateral extend of the exposure. Except for the Ro exposure where a clay layer was identified below the nummulitic accumulation, in all other sampling points the samples were collected from the nummulitic accumulation.

The calcareous nannoplankton assemblages were recovered from the sandy matrix present between the abundant *Nummulites* specimens in the nummulitic accumulation. The sample preparation was based on the gravity settling technique [2]. First, the sample represented by a rock fragment was crushed using a mortar and a pestle, and the equipment was carefully cleaned after this process to avoid the contamination. Then the obtained powder was placed in a beaker, distilled water was added, then it was stirred until a homogenous mixture was obtained and let to settle for 1-2 minutes. The supernatant was decanted into a second beaker and allow settling for 10 minutes and the settled fraction was used to prepare the smear slides. On every smear slide a minimum of 300 specimens were counted. Less calcareous nannoplankton rich samples were analyzed in 800 different fields of view. Calcareous nannoplankton taxa were classified according to online catalogue Nannotax 3 [4].

The smaller benthic foraminifera were recovered from the matrix present between the *Nummulites* specimens. The sandy samples were prepared in the laboratory following these steps: 1. weighing; 2. drying at 50 °C for 24 h; 3. weighing; 4. soaking in hydrogen peroxide solution with a concentration of 10%; 5. washing over a 63 µm mesh sieve; 6. drying the residue at 50 °C for 24 h; 7. dry-sieving using a 1 mm mesh sieve; 8. weighing the fraction less than 1 mm (and coarser than 63 µm); 9. (when appropriate) soaking the residue in a 3% hydrogen peroxide solution; 10. drying at 50 °C for 24 h; and 11. weighing the residue. The clayey samples were prepared using the standard micropaleontological method described also in Armstrong and Brasier [5]: drying at 50 °C for 24 h, weighing, boiling in a solution of tap water with one tablespoon of sodium carbonate added, for approximatively 1 hour, then washing over a 63 µm mesh sieve, drying at 50 °C for 24 h and weighing the residue. The residues were split into aliquots of about 200–300 specimens using a dry ASC Micro Sample Splitter and transferred onto a picking tray. The smaller foraminifera were picked under a binocular microscope (Optika, SZO-T+SZ-A1+SZ-ST8) using a fine paint brush (5/0) and stored and sorted on microslides. Identification was based on representative papers on the Eocene Tethyan regions [6,^7^,^8^,9]. Typical specimens were photographed and examined in detail with a scanning electron microscope (Hitachi 8230).

Raw calcareous nannoplankton and smaller benthic foraminifera species counts are shown in Tables 1 and 2 in [3], respectively. Counts of calcareous nannoplankton taxa, were converted into relative proportions, and data of those taxa which have more than ~2% relative abundance (Table 3 in [3]), were ARCSIN-normalized (Table 5 in [3]); counts of all smaller benthic foraminifera were converted into percentage data (Table 4 in [3]) and data on taxa having more than 1% relative abundance were ARCSIN-normalized (Table 6 in [3]). The micropaleontological data (for both calcareous nannoplankton and smaller benthic foraminifera) were normalized using MS Excel formula: ARSIN (SQRT(Relative abundance taxa/100)). Smaller benthic foraminiferal abundance data on species and groups (prepared sample weight, split value, count per split and per gram, count and percentage of agglutinated foraminifera, count and percentage of calcareous benthic foraminifera, count and percentage of infaunal and epifaunal species) and the calculated BFOI values are presented in Table 7 in [3]. Diversity indices (Taxa, Individuals, Dominance, Simpson 1-D, Shannon-H, Evenness_e^H/S^, Brillouin, Menhinick, Margalef, Equitability_J, Fisher_alpha, Berger-Parker, Chao-1 – explained in Hammer and Harper [10]) are shown in Table 8 in [3].

Hierarchical clustering using Ward's algorithm (distance) and the Euclidean similarity index (linkage) and Principal component analysis (PCA) were performed on both calcareous nannoplankton and smaller benthic foraminifera datasets; samples with less than 50 calcareous nannoplankton specimens and calcareous nannoplankton taxa with less than 2% relative abundance, and smaller benthic foraminifera taxa with less than 1% relative abundance were excluded from the datasets before the analysis. Principal component analysis (PCA) was applied to the data using a variance–covariance matrix.

These analyses (diversity, clustering and PCA) were performed using software package PAST 3.26.b [11].

## CRediT Author Statement

**Raluca Bindiu-Haitonic and Ramona Bălc**: Methodology, Investigation, Formal analysis, Writing – original draft; **Szabolcs-Attila Kövecsi:** Conceptualization, Investigation, Data curation, Visualization, Writing – original draft; **George Pleș:** Investigation, Writing – original draft; **Lóránd Silye:** Conceptualization, Writing – review & editing, Funding acquisition, Supervision, Validation, Project administration.

## Declaration of Competing Interest

The authors declare that they have no known competing financial interests or personal relationships which have, or could be perceived to have, influenced the work reported in this article.
